# Supplementary material for: Physical and Physiological Profiles of Aerobic and Anaerobic Capacities in Young Basketball Players
Source: Int J Environ Res Public Health. 2020 Feb 21;17(4):1409. doi: 10.3390/ijerph17041409 (PMC7068281; doi:10.3390/ijerph17041409)
Supplement: Supplementary file 1 [file ijerph-17-01409-s001.pdf]

SUPPLEMENTARY MATERIALS

Table S1. Descriptive results of the aerobic test grouped by gender and age.

			U´14		U´16		U´18	
			Mean	*ST*	*Mean*	*ST*	*Mean*	*ST*
MALE	Technical-tactical V.	Shots	10,40	1,150	12,12	1,10	11,81	0,87
		Scores	3,53	1,61	5,77	2,02	5,36	2,13
		Efficacy	33%	14%	47%	15%	45%	17%
	O. E. L. K. V. related to Distance	Parts of Circ.	127,56	13,991	143,48	13,05	139,85	9,39
		Distance (m)	1913,43	209,86	2152,25	195,85	2097,72	140,86
	Objective External Load Kinematics V. related to Accelerometry	Acc	235,90	97,32	250,83	74,97	236,90	81,23
		Decel	207	103,79	235,09	76,69	220,27	82,40
		Acc/min	19,37	8,41	20,85	6,24	19,71	6,79
		Decel/min	16,96	8,89	19,54	6,38	18,34	6,87
	Objective Internal Load V.	HR Med	183,28	8,75	179,03	8,71	173,48	8,71
		HR Max	194,43	7,07	191,96	7,57	188,21	8,85
		HR Rec	138,21	11,62	144,35	9,57	128,27	13,94
		% HR Max	91,56	4,80	89,5	4,31	86,74	4,35
	Objective External Load Neuromuscular V.	Impacts	311,31	209,07	231,03	237,60	330,63	262,90
		PlayerLoad	27,57	3,491	30,96	5,25	30,81	3,38
		PL/min	2,29	0,28	2,57	0,44	2,48	0,28
			**U´14**		**U´16**		**U´18**	
			Mean	*ST*	*Mean*	*ST*	*Mean*	*ST*
FEMALE	Technical-tactical V.	Shots	10,12	1,20	9,42	0,75	11,23	0,75
		Scores	2,62	1,02	3,42	0,93	4,47	2,49
		Efficacy	26,1%	9,9%	37%	12%	39%	21%
	O. E. L. K. V. related to Distance	Parts of Circ.	125,13	16,05	113,57	8,82	131,27	9,36
		Distance (m)	1876,87	240,80	1703,57	132,30	1969,09	140,50
	Objective External Load Kinematics V. related to Accelerometry	Acc	263,5	76,95	364,71	84,50	401,27	46,79
		Decel	249,5	56,16	338,14	84,12	401,18	46,83
		Acc/min	22,06	6,36	30,44	6,93	33,44	3,90
		Decel/min	20,89	4,64	28,23	6,91	33,42	3,90
	Objective Internal Load V.	HR Med	181,62	5,08	183,14	6,35	183,77	9,62
		HR Max	194,5	5,86	190,14	10,71	195,7	8,51
		HR Rec	145,25	10,90	135	5,87	133,63	13,07
		% HR Max	91,41	2,53	90,92	3,17	91,56	4,80
	Objective External Load Neuromuscular V.	Impacts	221,12	149,18	235,57	31,96	391,59	118,14
		PlayerLoad	25,29	3,07	21,92	3,85	26,86	2,34
		PL/min	2,10	0,25	1,83	0,31	2,23	0,19

O. E. L. K. V. related to Distance: Objective External Load Kinematics Variables related to Distance; Parts of Circ: Parts of Circuits; Acc: Accelerations; Decel: Decelerations; HR Med: Heart Rate Medium; HR Max: Heart Rate Maximum; HR Rest: Heart Rate Recovery; PlayerLoad/min: PlayerLoad/minute.

Table S2. Descriptive results of the anaerobic test grouped by gender and age.

			U´14		U´16		U´18	
			Mean	ST	Mean	ST	Mean	ST
MALE	Technical-tactical V.	Shots	27,44	3,48	29,56	2,86	29,43	2,49
		Scores	18,96	5,37	26,16	3,18	25,68	4,64
		Efficacy	80%	16%	80%	13%	81%	15%
	O. E. L. K. V. related to Distance	Parts of Circ.	109,26	14,39	115,80	10,56	115,14	9,55
		Distance (m)	819,44	107,92	868,50	79,17	863,57	71,59
	Objective External Load Kinematics V. related to Accelerometry	Acc	153,78	11,71	155,92	21,37	110,21	40,58
		Decel	131,30	16,33	144,00	15,11	104,93	44,48
		Acc/min	30,86	2,59	31,25	4,19	22,46	8,40
		Decel/min	26,27	3,20	28,87	3,06	21,39	9,18
	Objective Internal Load V.	HR Med	181,00	7,22	173,96	13,76	167,50	8,10
		HR Max	198,37	6,32	190,88	11,11	185,32	7,57
		HR Rec	159,63	7,80	144,68	12,79	133,00	12,98
		% HR Max	89,00	3,94	89,82	4,13	87,15	5,13
	Objective External Load Neuromuscular V.	Impacts	189,52	49,87	246,08	126,10	296,18	120,14
		PlayerLoad	16,77	4,59	17,97	6,52	14,89	3,98
		PL/min	3,35	0,92	3,59	1,30	2,98	0,80
			U´14		U´16		U´18	
			Mean	ST	Mean	ST	Mean	ST
FEMALE	Technical-tactical V.	Shots	29,00	1,90	27,86	2,03	29,95	1,30
		Scores	22,09	2,81	23,71	3,02	25,50	3,70
		Efficacy	87%	12%	91%	9%	81%	12%
	O. E. L. K. V. related to Distance	Parts of Circ.	111,09	6,87	108,57	7,44	116,20	5,05
		Distance (m)	833,18	51,49	814,29	55,79	871,50	37,87
	Objective External Load Kinematics V. related to Accelerometry	Acc	140,91	20,79	149,29	6,14	164,30	18,54
		Decel	134,00	13,86	148,43	6,32	161,75	20,62
		Acc/min	28,14	4,18	29,54	1,40	32,86	3,71
		Decel/min	26,76	2,81	29,37	1,33	32,35	4,12
	Objective Internal Load V.	HR Med	178,45	13,86	179,71	5,43	173,25	8,44
		HR Max	200,82	3,59	194,71	3,54	192,15	7,02
		HR Rec	142,64	12,75	153,43	8,52	133,25	12,04
		% HR Max	83,93	5,73	86,86	3,99	83,10	2,45
	Objective External Load Neuromuscular V.	Impacts	302,09	115,22	207,86	11,94	255,35	56,03
		PlayerLoad	16,50	3,90	11,48	1,57	11,40	0,58
		PL/min	3,30	0,78	2,30	0,31	2,28	0,12

O. E. L. K. V. related to Distance: Objective External Load Kinematics Variables related to Distance; Parts of Circ: Parts of Circuits; Acc: Accelerations; Decel: Decelerations; HR Med: Heart Rate Medium; HR Max: Heart Rate Maximum; HR Rest: Heart Rate Recovery; PlayerLoad/min: PlayerLoad/ minute.
